# Modeling Metastatic Colonization in a Decellularized Organ Scaffold‐Based Perfusion Bioreactor

**DOI:** 10.1002/adhm.202100684

**Published:** 2021-11-17

**Authors:** Maria Rafaeva, Edward R. Horton, Adina R.D. Jensen, Chris D. Madsen, Raphael Reuten, Oliver Willacy, Christian B. Brøchner, Thomas H. Jensen, Kamilla Westarp Zornhagen, Marina Crespo, Dina S. Grønseth, Sebastian R. Nielsen, Manja Idorn, Per thor Straten, Kristoffer Rohrberg, Iben Spanggaard, Martin Højgaard, Ulrik Lassen, Janine T. Erler, Alejandro E. Mayorca‐Guiliani

**Affiliations:** ^1^ Biotech Research and Innovation Centre (BRIC) University of Copenhagen (UCPH) Ole Maaloes Vej 5 Copenhagen 2200 Denmark; ^2^ Division of Translational Cancer Research Department of Laboratory Medicine Lund University Lund 22242 Sweden; ^3^ Department of Pathology, Rigshospitalet Copenhagen University Hospital Blegdamsvej 9 Copenhagen 2100 Denmark; ^4^ National Center for Cancer Immune Therapy (CCIT) Department of Oncology University Hospital Herlev and Department of Immunology and Microbiology University of Copenhagen (UCPH) Herlev Ringvej 75 Herlev 2730 Denmark; ^5^ Department of Oncology Centre for Cancer and Organ Diseases, Rigshospitalet Copenhagen University Hospital Blegdamsvej 9 Copenhagen 2100 Denmark; ^6^ Present address: Institute of Experimental and Clinical Pharmacology and Toxicology Medical Faculty University of Freiburg. Freiburg Germany

**Keywords:** cancer metastasis, experimental methods, extracellular matrix, specialized bioreactors

## Abstract

Metastatic cancer spread is responsible for most cancer‐related deaths. To colonize a new organ, invading cells adapt to, and remodel, the local extracellular matrix (ECM), a network of proteins and proteoglycans underpinning all tissues, and a critical regulator of homeostasis and disease. However, there is a major lack in tools to study cancer cell behavior within native 3D ECM. Here, an in‐house designed bioreactor, where mouse organ ECM scaffolds are perfused and populated with cells that are challenged to colonize it, is presented. Using a specialized bioreactor chamber, it is possible to monitor cell behavior microscopically (e.g., proliferation, migration) within the organ scaffold. Cancer cells in this system recapitulate cell signaling observed in vivo and remodel complex native ECM. Moreover, the bioreactors are compatible with co‐culturing cell types of different genetic origin comprising the normal and tumor microenvironment. This degree of experimental flexibility in an organ‐specific and 3D context, opens new possibilities to study cell–cell and cell–ECM interplay and to model diseases in a controllable organ‐specific system ex vivo.

## Graphical abstract

Modeling complex diseases is crucial in order to understand their mechanisms and advance toward successful treatments. Here, a cancer metastasis model that replicates the intricate relationship between cells and a fundamental part of their environment, the extracellular matrix is shown. This model allows to follow cells as they colonize an organ scaffold, where they replicate signaling pathways and refashion their niche.

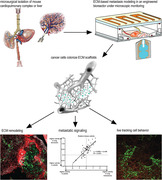

## 1 Introduction

The extracellular matrix (ECM) is a 3D network composed of collagens, glycoproteins, and proteoglycans that holds tissue structure in place. The ECM is an active regulator of tissue homeostasis, through the stiffness and elasticity of its structure,^[^ ^1^ ^]^ by presenting ECM ligands to cell receptors,^[^ ^2^ ^]^ or presenting cytokines and growth factors.^[^ ^3^ ^]^ When tissues are challenged by tumor development, the ECM is dramatically altered in ways that can support or suppress disease progression.^[^ ^4^ ^]^ Multiple players are involved in this process, such as cancer cells, fibroblasts, and immune cells.^[^ ^5^ ^]^ Linearized, cross‐linked fibrillar collagen is one of the ECM changes involved in cancer progression,^[^ ^6^ ^]^ but full understanding of complex ECM remodeling, especially in metastatic niches, is still lacking.^[^ ^7^ ^]^ The ECM is dynamically rebuilt, deposited, and degraded, releasing growth factors and signaling molecules, thus changing single cell response, such as migration,^[^ ^8^ ^]^ proliferation, and survival.^[^ ^5^, ^9^ ^]^

Native ECM extracts (collagen I, basement membrane) and various synthetic 3D systems that allow the modulation of ECM‐like parameters (e.g., stiffness, porosity, and ligand distribution)^[^ ^10^ ^]^ are used to model the ECM influence on cancer progression. While these approaches are more advanced than 2D systems, they still lack critical factors: 3D ECM architecture, complex native ECM composition, and the mechanical tension from constant tissue perfusion. Organ‐on‐chip technology combined with 3D printing has a potential to overcome most of these limitations,^[^ ^11^ ^]^ although recapitulating the organ‐specific and intricate interplay between cells and the ECM remains a challenge. Progress in determining the role of the specific host organ, or “soil,” on the behavior of disseminated cancer cell, or “seeds” aiming to colonize that organ, is hindered by a lack of model systems that accurately recapitulate native organ structure.

Organ decellularization is a source of native ECM.^[^ ^12^ ^]^ In this approach, cells are chemically removed leaving behind an insoluble, dimensionally intact ECM scaffold.^[^ ^13^ ^]^ Several recent studies suggest the use of decellularized tissues in cancer research to identify new genetic drivers,^[^ ^14^ ^]^ or find and validate targets for preclinical drug testing.^[^ ^15^ ^]^ These examples include use of tissue‐specific native ECM, or of processed matrix lacking original structural arrangement.^[^ ^16^ ^]^ In addition, decellularized scaffolds contain native altered ECM and, thus, provide in vivo cues influencing cell behavior.^[^ ^17^ ^]^

The crucial and often irreversible step in metastatic progression is colonization—an outgrowth of cancer cells in a new site after extravasation.^[^ ^18^ ^]^ Modeling metastatic colonization in a 3D tissue structure that reflects in vivo microenvironmental cues and their alterations is an outstanding challenge in cancer research.^[^ ^19^, ^20^ ^]^ Here, we present an in‐house designed model system based on a culture chamber tailored to house a decellularized, catheterized, mouse ECM scaffold. The chamber allows live microscopic monitoring, opening the way to study collective cell behavior. When grown in this 3D environment, cancer cells mimic in vivo metastatic kinase signaling and cancer‐associated ECM remodeling. Our system therefore advances the modeling of homeostasis and disease in a way that was not previously possible ex vivo.

## 2 Results

### 2.1 An ECM‐Based Bioreactor Designed for Modeling Metastatic Colonization

**Figure 1:**
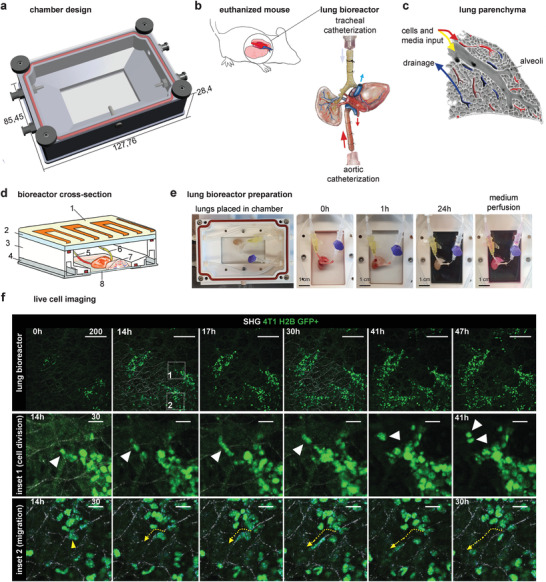
Design of decellularized organ scaffold‐based perfusion bioreactor and population with cancer cells. a) Chamber design schematic (dimensions in mm, cf. Figure S2, Supporting Information). b) Schematic for microsurgery and catheterization of one lung lobe for perfusion decellularization and introduction of cells. A lung lobe is excised along with the heart and catheterized through the aorta and the trachea. c) Schematic of lung liquid supply and drainage routes. d) Bioreactor chamber cross section showing: 1) heating element placed above or below the chamber, 2) transparent plastic lid, 3) chamber body, 4) metal chamber base, 5) aortic catheter, 6) tracheal catheter, 7) organ, and 8) optical glass window. e) Timeline of the lung lobe decellularization inside of a bioreactor chamber. Times shown since beginning of perfusion (deionized water 15 min, 0.5% DOC 24 h, medium perfusion before introducing cells). f) Live‐cell imaging of the lung bioreactor after population with 4T1‐H2B‐GFP breast cancer cells via trachea, monitored for 2 days. Simultaneous imaging of SHG (fibrillar collagens) and GFP signal (nuclei). Inset 1 shows a cell division event; inset 2 shows cell migration along collagen fiber (all scales in microns). See Movie S1, Supporting Information.

The main aim of this project is to design a method to investigate metastatic colonization in a 3D environment. This environment should provide a measure of in vivo complexity while allowing experimental flexibility and live monitoring, isolating single cell type–ECM interactions. Therefore, we designed a culture chamber (**Figure** **1**) that provides a sterile, airtight environment to a catheterized ECM scaffold. The chamber is equipped with connectors that ensure medium flow regulation and drainage, fitted with an optical window and made to fit in a microscope stage and enable monitoring of cell behavior (Figure 1b). As the mouse is the main model organism, we designed microsurgical operations to generate murine organ scaffolds for cell introduction, adapting procedures based on the In Situ Decellularization of Tissues method (ISDoT)^[^ ^13^ ^]^ (Figure S1, Supporting Information). As the first organ, we focused on the lung—a common site of metastasis.^[^ ^21^ ^]^ Our approach provides access to the circulation of mouse lung lobes excised from a euthanized mouse body (Figure 1b,c). In the lungs, catheterization of the trachea opens a route to the alveolar parenchyma via the bronchial tree and retrograde catheterization of the aorta leads to the pulmonary venous circulation via the left ventricle and atrium. These entrances can be used to introduce cells in distinct compartments and lead to a drainage route through the arterial circulation (Figure 1b,c; Figure S1, Supporting Information).

The organ segment, catheterized and excised en bloc, is placed in the chamber (Figure 1e; Figure S2, Supporting Information). For perfusion with reagents and cell culture medium the chamber is connected to liquid delivering and draining tubing controlled by a peristaltic pump. Temperature for cell culture conditions can be regulated by using a temperature control unit placed under or above the chamber.

The organ is first perfused with deionized water, then decellularized with 0.5% sodium deoxycholate (DOC) and washed with phosphate‐buffered saline (PBS) (Figure 1e). The lung lobe becomes translucent after 2 h (Figure 1e) and is decellularized after 24 h of the detergent perfusion (the process can be inspected through the transparent chamber lid), which can be confirmed at histological level (Figure S3a, Supporting Information). Next, the organ scaffold is perfused with cell culture medium and equilibrated at 37 °C before a cell suspension is slowly injected into the tubing and perfused into the scaffold. ECM scaffolds generated by this approach retain their ECM composition and 3D architecture.^[^ ^13^ ^]^

First, we explored if 4T1 cells, a triple‐negative mouse breast cancer cell line that metastasizes in vivo to the lung, would colonize perfused lung scaffolds. We perfused histone H2B‐GFP‐tagged 4T1 cells (nuclei labeled) into the lung ECM bioreactor via the trachea and used two‐photon (2P) confocal microscopy to visualize cells simultaneously with fibrillar collagens by second harmonics generation (SHG) imaging (Figure 1f; Movie S1, Supporting Information). Live‐cell imaging for 48 h demonstrated lung ECM colonization by 4T1 cells, including proliferation (Figure 1f, inset 1), invasion, and migration along collagen fibers (Figure 1f, inset 2). Interestingly, the migration of cancer cells along collagen fibers seen here is similar to previous observations recorded by intravital imaging of in vivo tumors.^[^ ^22^ ^]^

### 2.2 Cancer Cells Remodel the Native ECM after Traversing Vascular Basement Membranes

**Figure 2:**
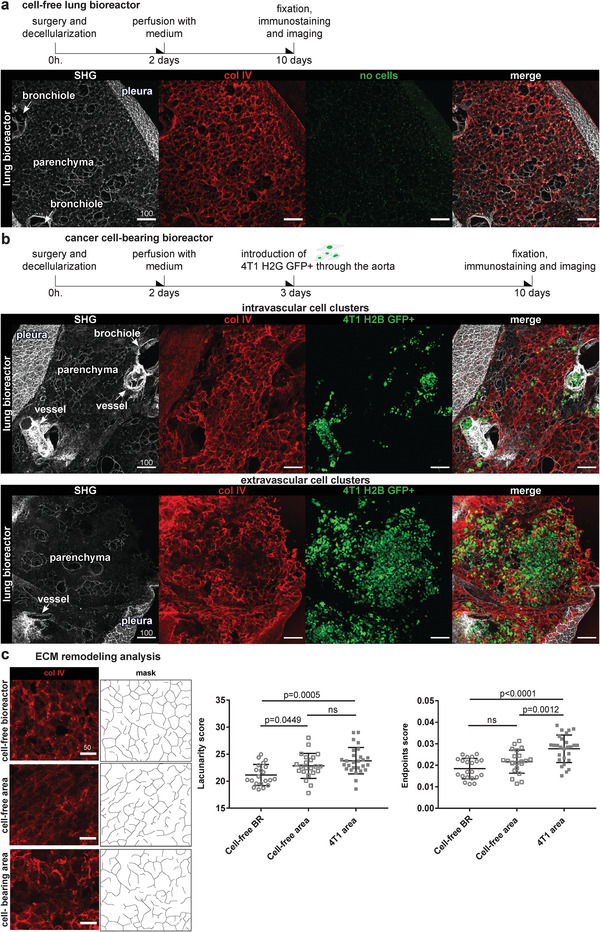
Cancer cells colonize the lung scaffold through the aortic route and remodel the ECM scaffold. a) Cell‐free bioreactor sample imaged for fibrillar collagens (by SHG) and collagen IV. b) 4T1‐H2B‐GFP populated bioreactor sample imaged for fibrillar collagens and collagen IV. Representative images of “intravascular cell clusters” (cells contained in the vessels) and “extravascular cell clusters” (cells colonizing parenchyma). *n* = 3 bioreactors, see Figure S4, Supporting Information for more examples. c) Quantitative analysis of ECM remodeling. Left panel: representative images and derived masks of regions of interest (ROI) of col IV‐stained lung parenchyma from cell‐free control bioreactor, 4T1 bioreactor in cell‐free area, and in area with an extravascular cluster of 4T1s. Right panel: quantification of lacunarity (irregularity of patterns) and endpoints (endings of ECM branches) from col IV created masks by TWOMBLI plugin. Data points present values per ROIs, Mean ± SD. *n* = 4 bioreactors with 4T1, *n* = 3 control bioreactors (BR). *p* values are calculated by one‐way ANOVA test. All scales in microns.

We next sought to introduce 4T1 cells through the aortic route in order to force them to migrate through vascular basement membranes (BMs). We previously showed that BM integrity is maintained in ISDoT decellularized lungs.^[^ ^13^ ^]^ To discriminate between parenchymal and vascular cell localization, we first characterized the 3D organization of lung ECM scaffold by immunostaining against collagen IV. Fluorescent collagen IV along with SHG imaging revealed BMs and fibrillar collagens, respectively. These major lung ECM components allow to identify alveolar parenchyma, vessels, airways, and pleura^[^ ^23^ ^]^ (**Figure** **2**a,b). After injecting 4T1‐H2B‐GFP cells through the aorta (Figure 2b) we observed that, within first hours and days of injection, cells were contained within the vascular bed. We termed this “intravascular” localization. After 1 week of culturing, 4T1‐H2B‐GFP cells were found both inside the vascular bed and within the parenchymal tissue (Figure 2b; Figure S4, Supporting Information), demonstrating that cells migrated through the vascular BM and colonized the adjacent parenchyma; we termed this “extravascular” localization. When 4T1‐H2B‐GFP cells are contained intravascularly, the surrounding parenchymal ECM appeared anatomically normal (as in the control/cell‐free bioreactor staining). Interestingly, when cells formed clusters in the parenchyma, we observed changes in the BM structure as shown with col IV (Figure 2b; Figure S4a,b, Supporting Information). Clinical pathologists examined this imaging and confirmed the abnormal histology of the ECM characterized by the septal structure destruction in the areas with “extravascular” cell clusters and a clear reduction in col IV, described as “erasing” (Figure 2b; Figure S4a,b, Supporting Information). We performed ECM image analysis based on col IV staining and found cancer cells bearing bioreactor to have increased lacunarity (i.e., irregularity in ECM organization). In addition, in the areas invaded by cell clusters we detected an increased number of endpoints in the ECM structure (i.e., interrupted alveolar septa) (Figure 2c). This result shows that cancer cells introduced via the aorta can cross vascular BMs, colonize and remodel ECM of decellularized lung scaffolds in the perfusion bioreactor.

### 2.3 Cancer Cells in the Lung Bioreactor Recapitulate In Vivo Metastatic Signaling

**Figure 3:**
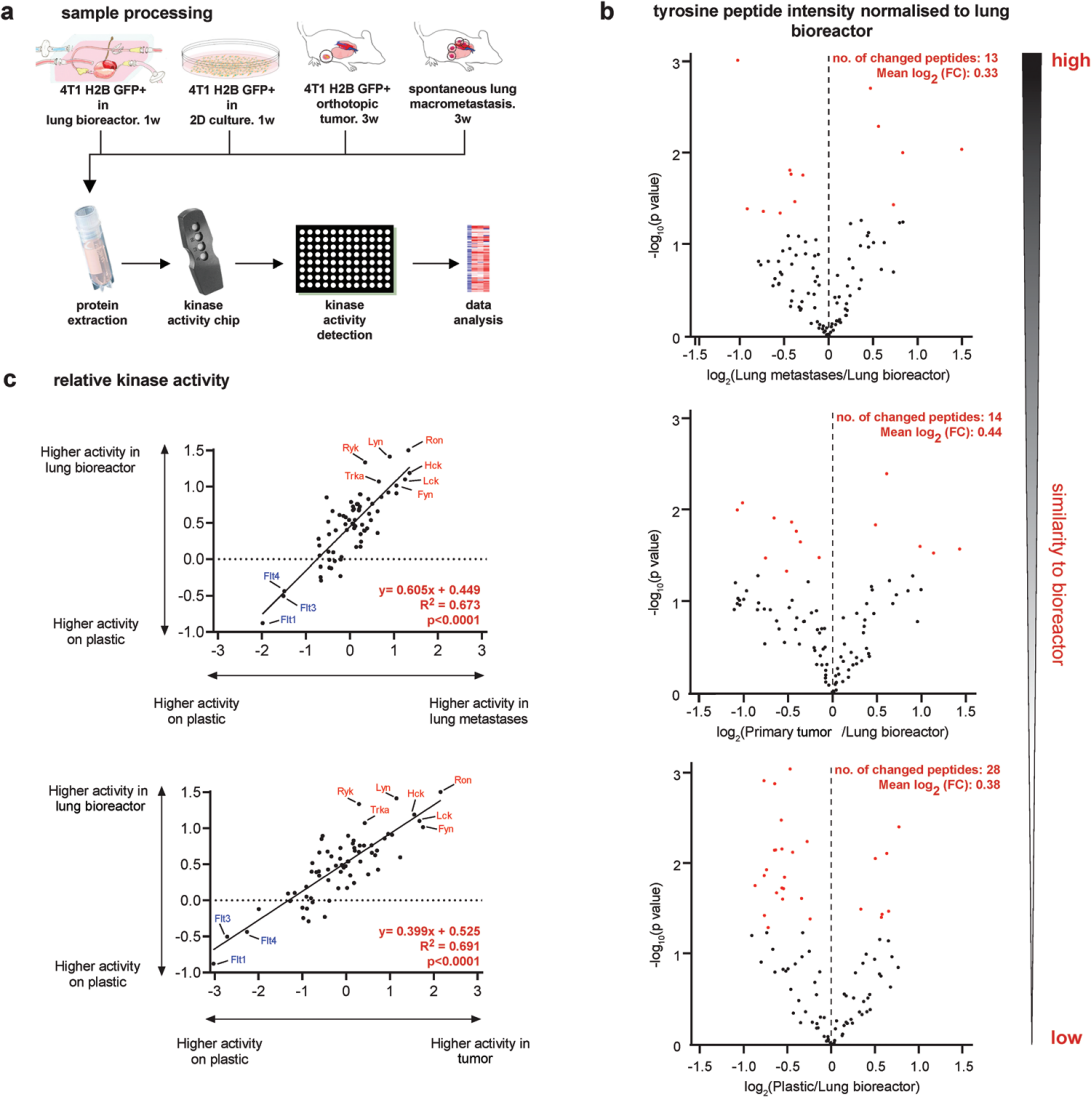
Cancer cells in the lung bioreactor recapitulate in vivo metastatic tyrosine kinase signaling. a) Schematic showing sample preparation and processing. b) Volcano plots showing that lung bioreactors have the least significant changes (number of changed peptides, mean fold change—FC) of peptide intensities relative to lung metastases, followed by primary tumors and cells cultured on plastic. Significantly changed values (unpaired two‐tailed *t‐*test, *p* < 0.05) are in red. c) Positive correlation of the relative tyrosine kinase activity (mean kinase statistic values) between 1) 4T1‐H2B‐GFP lung bioreactor and lung metastasis (normalized to culture on plastic). Simple linear regression analysis: *R* ^2^ = 0.673, *p* < 0.0001; 2) 4T1‐H2B‐GFP lung bioreactor and primary tumor (normalized to culture on plastic). Simple linear regression analysis: *R* ^2^ = 0.691, *p* <0.0001. *n* = 3 biological repeats for all conditions, except *n* = 2 for primary tumor. Top upregulated kinases are highlighted in red (mean kinase statistic > 1 in lung bioreactor) and top downregulated (mean kinase statistic < −0.4 in lung bioreactor) in blue. For dataset see Tables S1, S2, Supporting Information.

We were interested in studying whether culturing of cancer cells inside bioreactor is able to change their signaling and recapitulate signaling profile in metastatic lung in vivo. We focused on signaling pathways involving tyrosine kinases since they are stimulated by ECM‐mediated adhesion^[^ ^24^ ^]^ and are promising therapeutic anti‐cancer targets with many inhibitors in clinical use.^[^ ^25^ ^]^ Here, we probed lysates from 4T1‐H2B‐GFP cancer cells cultured in a lung bioreactor or grown on plastic, as well as from orthotopic 4T1‐H2B‐GFP primary tumors and spontaneous lung metastases (**Figure** **3**a) using Pamgene kinase activity profiling (Pamgene, Netherlands).^[^ ^26^ ^]^ Analysis of phosphorylation levels of peptides showed lowest number of peptides and smallest magnitude of change between the lung bioreactor and in vivo lung metastases (Figure 3b; Table S1, Supporting Information) comparing to other conditions.

Using a prediction tool to infer the activity of upstream tyrosine kinases,^[^ ^26^ ^]^ we show that changes in kinase activity in lung bioreactor positively correlate with altered kinase activity observed in in vivo lung metastases and primary tumor (Figure 3c; Table S2, Supporting Information). Among the most upregulated kinases (>1 mean kinase statistic in bioreactor) are Ron, or Macrophage‐stimulating 1 receptor kinase (MST1R), Lck, Hck, and Fyn kinases; while most downregulated (<−0.4 in bioreactor) are Flt 1, 3, 4 kinases. Ron receptor kinase is known to be overexpressed in cancer and to promote metastatic phenotype by epigenetic reprogramming,^[^ ^27^ ^]^ which also makes it a predictive marker for patient's survival.^[^ ^28^ ^]^ Lck and Hck are Src tyrosine kinase family members usually expressed in immune or hematopoietic cells, but recently also described to be expressed in cancer cells.^[^ ^29^, ^30^ ^]^

Remarkably, this result suggests that culture in native ECM impacts kinase activity regulation, that is, cell signaling, providing cues that make cancer cell signaling approach the in vivo situation.

### 2.4 Bioreactor Serves as an ECM‐Based Platform for Studying the Interaction between Multiple Cell Types

**Figure 4:**
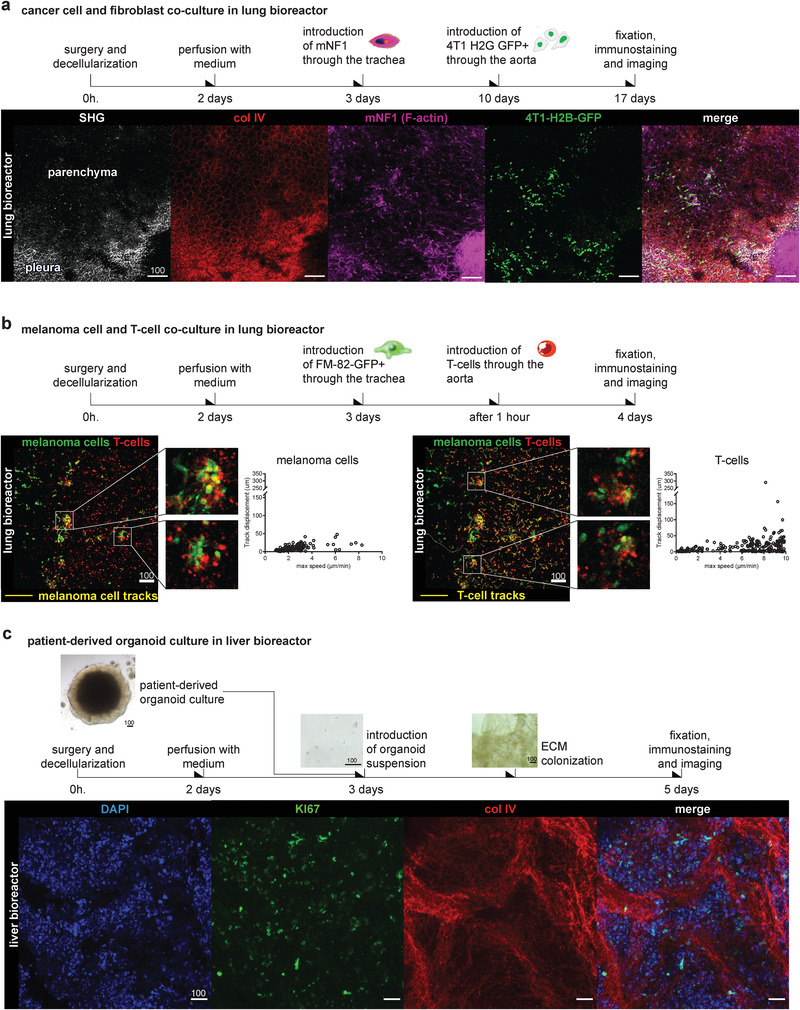
Lung bioreactor as a platform for culturing multiple cell types. a) mNF1 and 4T1‐H2B‐GFP co‐culture bioreactor imaged for fibrillar collagens and collagen IV. See Figure S6, Supporting Information for an additional example. b) Cell tracking of human melanoma cells (FM‐82‐GFP) and primary T‐cells after 24 h of co‐culture. Insets show melanoma cells covered with T‐cells. Tracks are shown in yellow. Graphs present track displacement and maximum speed (µm min^−1^) of 148 cancer cell migration tracks and 370 T‐cell migration tracks, respectively. Automated tracking performed for a period of 2 h. *n* = 1 bioreactor. c) Liver bioreactor populated with patient‐derived organoids cells from colorectal cancer metastasis. Organoids were pre‐conditioned for 24 h in medium without growth factors and cultured in the same conditions for 48 h post‐injection. DAPI shows cell nuclei, ki‐67 staining shows nuclei of proliferating cells. All scales in microns.

As the tumor microenvironment is comprised of several cell types, we applied lung bioreactors for modeling cell interactions in a 3D ECM scaffold. Introducing cells through different delivery routes in the lung can serve as an advantage allowing to imitate stromal population and metastatic colonization. First, we introduced normal murine mammary fibroblasts mNF1 through the trachea and evaluated extent of scaffold population (Figure S5, Supporting Information). Within 1 week, fibroblasts fully populated the parenchyma and pleura of the scaffold without altering the col IV structure in the alveolar parenchyma, in contrast to what we found for the “extravascular” cancer cell clusters. Fibroblasts spread along alveolar septa or stayed more nodular (Figure S5, Supporting Information). We next injected 4T1‐H2B‐GFP cells through the aorta into bioreactors populated with mNF1 and co‐cultured them for 1 week. Imaging of whole‐mount samples showed multiple areas of interaction between cell types, both inside parenchyma and on the pleura (**Figure** **4**a, Figure S6, Supporting Information).

We next tested if the bioreactor can serve as a platform for studying dynamic cell interactions, such as targeted T‐cell attack of cancer cells. As example, we co‐cultured human melanoma cells FM82‐GFP‐Luc with genetically engineered human T‐cells, carrying melanoma‐associated antigen 3 (MAGE‐A3^a3a^) T‐cell receptors (TCRs).^[^ ^31^ ^]^ Cancer cells were injected through the trachea, to populate the parenchyma and 1 h later dye‐labeled T‐cells were introduced through the aorta. Analyzing live‐cell imaging movies we found T‐cells to cover almost immotile cancer cell clusters and single T‐cells actively migrating in the scaffold after 24 h (Figure 4b). Only a few T‐cells showed unidirectional persistent migration (track displacement > 100 µm) (Movie S2, Supporting Information). Therefore, T‐cells survived and successfully found clusters of cancer cells within the native organ ECM scaffold.

Further, we extended the application of our in‐house bioreactor set‐up to another metastatic target organ, the liver. In this case, surgery was optimized to excise one liver lobe along with its portal vascularization and place it into the bioreactor chamber (Figure S7a,b, Supporting Information). Here, the portal vein directly leads to the portal spaces of the liver parenchyma during cell population (Figure S7a, Supporting Information). This entrance provides a drainage route through the arterial circulation and biliary ducts. The liver decellularization takes longer than the lung—up to 72 h until the lobe appears translucent and cells are removed (Figure S3b, Supporting Information).

Pancreatic cancer cells frequently metastasize to the liver and their survival was shown to be supported by macrophages in this new niche.^[^ ^32^ ^]^ We tested if the liver bioreactor can be colonized with mouse pancreatic cancer cells and primary macrophages by sequential introduction of them via the portal vein. We found that liver parenchyma was populated with both cell types within 24 h (Figure S8, Supporting Information) and, thus, these cell types can survive and be applied in our liver bioreactor.

In addition, colorectal cancer cells primarily metastasize to the liver and recent advances in organoid technology allowed to establish patient‐derived liver metastases as in vitro expanding 3D cultures of cancer cells (Figure S9a, Supporting Information).^[^ ^33^ ^]^ They are used to identify personalized treatments,^[^ ^34^ ^]^ however, they lack a native microenvironment given they are routinely cultured in mouse‐derived basement membrane extract (Matrigel), often with excessive growth factor supply.^[^ ^35^ ^]^ A liver bioreactor can offer an organ‐specific microenvironment, and we tested the survival of patient‐derived metastatic colorectal cancer organoids in our system. Given this model would be relevant for drug testing, which is usually performed without growth factors, we first performed cell population and culturing in these conditions. After pre‐culturing of the organoids without growth factors for 24 h, disaggregation into a cell suspension and introduction via the portal vein (Figure 4c), we cultured them in the liver ECM scaffold for 48 h. We observed proliferating cells (revealed by ki67+ staining), although they had not yet occupied the parenchyma (seen partially contained) (Figure 4c). To confirm that organoids‐derived cells can populate parenchymal ECM, we introduced cell suspension and cultured cells for 1 week in the presence of growth factors. Imaging confirmed evenly distributed single cells in a liver scaffold (Figure S9b, Supporting Information).

In summary, these examples show that both lung and liver can be used in our bioreactor set‐up for culturing cell combinations and primary patient‐derived cells, which can be followed by live‐cell imaging or whole mount sample imaging at fixed time points.

## 3 Discussion

The ECM is a major regulator of cell behavior, and yet there is a lack of tools to study the relationship between cells and native ECM in isolation. Here, we present a novel method to populate native ECM scaffolds with preserved 3D architecture by repurposing our ISDoT perfusion‐based mouse organ decellularization.^[^ ^13^ ^]^ We show that cell behavior inside scaffolds can be monitored by live‐cell imaging or end‐point analysis after immunostaining of whole mount samples (Figures 1, 2), or by kinase activity profiling (Figure 3). Metastatic kinase signaling and ECM remodeling occur in our cancer cell bioreactors, recapitulating in vivo metastasis (Figures 2, 3). The bioreactors provide the conditions for the co‐culture of normal cells of different origin as well as culture of patient‐derived organoids (Figure 4).

This system overcomes experimental limitations inherent to in vivo models. While a few studies have described cell introduction into the rat or mouse lung and liver ECM scaffolds by perfusion (via trachea for the lung, portal vein for the liver),^[^ ^36^, ^37^ ^]^ to our knowledge, this is the first decellularized organ scaffold‐based perfusion bioreactor designed with the possibility of live‐cell imaging combined with ECM visualization. This opens the way to study the events that lead to metastatic colonization, as well as single cell and collective behavior in a metastatic target. This also provides an opportunity to gain dynamic information on how cells interact with the native ECM in the normal, “healthy” organ ECM, as well as abnormal, “diseased” ECM.

Our results suggest that culturing only cancer cells in a healthy ECM scaffold closely mimics cell signaling profile observed in vivo (Figure 3). This can be a reflection of 3D growth, and of the scaffold features, such as signaling from the ECM itself or retained factors. Earlier studies suggested that 3D versus 2D growth of cancer cells affects cell signaling, with 3D being more relevant for studying drug response.^[^ ^38^ ^]^ Next, it remains to be established if tissue‐specific ECM composition and architecture are playing additional role in reconstructing in vivo conditions.

Our bioreactor demonstrates that the complex native organ ECM can be remodeled by the colonizing cancer cells. In particular, cancer cells were able to alter alveolar septa and degrade collagen IV, which we previously saw to be downregulated in the 4T1 metastatic lung proteomics analysis.^[^ ^13^ ^]^Exposing cancer cells to the native organ ECM challenges them to overcome ECM barriers and occupy niches, as they do in vivo. Importantly, whole mount sample staining and analysis enables visualization and assessment of ECM 3D organization and how cells interact with a complex environment. Using this approach to map structural arrangement of ECM components will be beneficial for development of tissue‐specific ECM mimics in the future.

The presence of the cancer cell clusters localized in intra‐ and extravascular areas at the same time suggests that crossing of vascular BM by cells is not simultaneous. Multiple factors can be responsible for this observation, such as different proteolytic activity of the cells,^[^ ^39^ ^]^ the heterogeneity of BMs inside of the organ, its mechanics,^[^ ^40^ ^]^ or a combination of those.^[^ ^41^ ^]^ Our approach can help in addressing this question in an organ‐specific context, which is not possible with available in vitro assays (gelatin degradation, organotypic culturing, etc.).

We did not identify ECM alterations by culturing normal fibroblasts in the lung parenchyma (Figure S5, Supporting Information) using our approach for the ECM visualization (SHG and collagen IV antibody staining). This could be fibroblast type‐specific, and may change with culturing cancer‐associated fibroblasts, major ECM producers and remodelers in tumor microenvironment.^[^ ^42^ ^]^ Native ECM could be remodeled by cells in the process of deposition of newly synthesized ECM and recent techniques of labeling nascent ECM by incorporating azide‐modified amino acids^[^ ^43^ ^]^ can help to distinguish newly deposited ECM from the original scaffold.

Finally, we show the bioreactor's versatility by culturing cells which have different genetic backgrounds than the ECM scaffold and open room for translational applications of this system. Modeling immunotherapy in an organ‐specific ECM can further address the role of the matrix architecture in T‐cells distribution^[^ ^44^ ^]^ and, by adding other cell types, address specificity of T‐cells targeting. Recent development of the organoids field supported personalized treatment of cancer patients and allowed to preserve genetic heterogeneity of cancer cells in vitro.^[^ ^34^, ^45^ ^]^ Given organoid‐derived cells successfully populated bioreactor, we suggest utilizing it as an alternative environment for testing organ‐specific therapy response.

As mouse‐derived ECM extracts are widely used for establishing and propagating organoids,^[^ ^35^ ^]^ differences between mouse and human ECM are usually neglected. Indeed, composition of the structural ECM is known to be well conserved among these species,^[^ ^46^ ^]^ with differences attributed to few amino acids in specific peptides.

Importantly, our bioreactor set‐up is not restricted to cancer studies and could be used to study other lung or liver‐specific diseases related to changes in the ECM. The main limitations of our approach are the lack of high‐throughput read‐outs, predefined nature and variability of decellularized tissue, and the requirement for advanced surgical skills and special equipment.

## 4 Conclusion

In summary, we present a powerful platform to study cells within a native ECM microenvironment under controlled conditions, which allows us to assess both their response to and their influence on the organ‐specific ECM. We focus here on the application to model metastatic cancer, highlighting the possibility to monitor critical cell behavior and the interaction of several cell types. Our approach creates opportunity to model elements of normal homeostasis and disease progression ex vivo.

## 5 Experimental Section

### Mouse Work

8‐weeks‐old female BALB/c and C57BL/6 mice were purchased from Taconic. For generating bioreactors, BALB/c mice were used for seeding 4T1‐H2B‐GFP cells and/or mNF1 fibroblasts; C57BL/6 mice were used for seeding primary macrophages and KPCmT4 cells. Seeding with human cells (FM82‐GFP‐Luc and primary T‐cells, or cells from patient‐derived cancer organoids) was done in BALB/c‐derived scaffolds.

For generation of samples for kinase profiling, BALB/c mice were orthotopically injected with 5 × 10^5^ 4T1‐H2B‐GFP cells in a mammary fat pad. Tumors were surgically resected when reaching the humane endpoint (10 mm diameter) and snap frozen. The mice were maintained alive to develop macroscopic lung metastases and sacrificed 3 weeks after the initial resection. Metastatic areas were dissected and snap frozen. All experiments were carried out under authorization and guidance from the Danish Inspectorate for Animal Experimentation (permission number #2017‐15‐0201‐01265).

### Bioreactor Chamber Fabrication

A detailed schematic of the bioreactor chamber can be found in Figure S2, Supporting Information. The chamber was fabricated from blocks of material on a digitally controlled milling machine by Ebers Medical (Zaragoza, Spain). It consisted of a body, a base, and a lid. The body frame, made from white biocompatible polypropylene, had six entrances with inserted luer connectors (Nordson Medical, USA) on the sides and grooves for silicone o‐rings (Barnwell, UK) on the top, for creating airtightness. The base, made from anodized aluminum, had a central window fitted with an optical glass (Corning, USA) of 75 × 50 mm^2^ size and 1 mm thickness. The transparent polycarbonate lid was tightened to the body with medical nylon screws, while the base was attached to the body by steel screws. All chamber materials were thermostable and autoclavable.

Perfusion was carried out with a peristaltic pump (Ole Dich, Denmark). Solutions were supplied through silicone tubing (Ole Dich, Denmark) of 1.5 mm in diameter. Chamber drainage was ensured by tubing with a double‐size internal diameter of 3 mm. The drainage tube could be placed to allow a level of liquid of ≈5 mm depth. To keep sterile conditions, the chamber and tubing were thoroughly washed (with 0.2% acetic acid solution and distilled water) and autoclaved before use. For assembling chamber parts were kept under a LAF bench. Perfused solutions were kept sterile and airtight, as well as a bottle for liquid waste from the drainage.

### Lung ECM Scaffold Generation

A photo sequence of the operation is available in the Figure S1, Supporting Information. Detailed surgical protocols for mouse microsurgery are previously published.^[^ ^47^ ^]^ Mice were euthanized as described, by suffocation with CO_2_, then shaved with a clipper (Oster, USA) and disinfected with 70% ethanol. The mouse was pinned to a polystyrene tray in the supine position and put under a surgical microscope (Leica S6D, Leica, Germany). A skin incision along the vertical axis from pelvis to hyoid bone allowed to expose the peritoneal and thoracic walls. Incisions through the sixth intercostal spaces and sternotomy revealed the cardiopulmonary complex. The incision was extended superiorly, cutting through the connective tissue between the submandibular salivary glands and the infra‐hyoid musculature to expose the trachea and the thyroid cartilage. Next, the descending cava vein was cauterized with a bipolar cautery (Minicutter, KLS Martin, USA) and the esophagus was sectioned. The connective tissue binding the lungs to the esophagus and diaphragm was carefully sectioned.

Blunt dissection of the thymus exposed the major vessels. To focus the flow into the cardio‐pulmonary complex, the following arteries were ligated and sectioned: left subclavian, left common carotid, and brachiocephalic. In addition, brachiocephalic veins were cauterized and sectioned. The cricothyroid ligament was cut and a 24G catheter (BD, USA) was introduced into the trachea until the tip of the catheter reached the bronchial bifurcation (approximately located behind the aortic arc). It was secured with four stitches around the trachea (6‐0 vicryl, Johnson and Johnson, USA). Next, the descending aorta was cauterized at the height of L1 vertebra and then the mouse was sectioned in half. The aorta was catheterized with a 27G catheter (Terumo, Japan), the tip reaching the aortic arc. The catheter was secured with four micro‐stitches (9‐0 vicryl, BBraun, Spain).

Four of the five pulmonary lobes were resected. The choice of the remaining lobe depends on the operator's preference. Here, was chosen the right superior lobe. The other lobes were resected in a clockwise sequence beginning with the left, then post‐caval, inferior, and middle lobes. Each lobe was excised by ligating the arteries, veins, bronchial branches and then excising with a bipolar cautery. The aorta was separated from the pre‐vertebral plane, then the cardiopulmonary complex, and finally the trachea from its attachments. The quality of the perfusion was tested by slowly injecting PBS into the catheter. The aorta was then moved superiorly, and organ construct was transferred en bloc to the bioreactor chamber base.

### Liver ECM Scaffold Generation

The mouse was euthanized and prepared as specified above and the lower part of the mouse separated as previously described.^[^ ^47^ ^]^ The peritoneum was sectioned along the midline, extending the incision to expose the liver (Figure S7, Supporting Information). Then, the mouse was rotated to face the diaphragm. Using a Metzembaum curve scissors, a cut between the thoracic wall and the diaphragm was made and the scissor was passed between the diaphragm and the liver, parallel to the posterior thoracic wall and in front of the spine, going beyond the midline. The mouse was rotated again to face the peritoneum. The liver was delicately elevated to expose its ventral face while sectioning the ligaments attaching the hepatic lobes to the digestive tract; this will expose the intestines, which must be elevated to the right to expose the portal vein. The portal vein was catheterized with a 27G catheter (Terumo, Japan) until the tip of the catheter penetrated the left hepatic lobe 3 to 5 mm. To secure the catheter, a 6‐0 suture was passed behind the left lobe, placed around the entrance of the portal branch into the lobe and tightened with a stitch. This stitch locked the flow into the lobe. Three additional stitches along the portal vein immobilized the catheter. The remaining lobes were discarded. Then the catheterized portal vein and the left hepatic lobe were excised. The quality of the perfusion was tested by slowly injecting PBS into the catheter (the hepatic lobe should blanch).

### Decellularization

Organ lobes were carefully placed into a bioreactor chamber filled with 5 mL of ice‐cold PBS under a LAF bench. Luer connectors in the chamber wall were filled with PBS to diminish the amount of air in the perfusion. Using sterile instruments, the catheters were attached to the Luer connectors and a lid was attached and fixed on a chamber. Deionized water was perfused for 15 min at flow speed of 150 µL min^−1^ to remove blood. Decellularization was achieved with 0.5% DOC (Sigma, USA) perfusion for 24 h (for the lung) or up to 72 h (for the liver) at flow speed 150 µL min^−1^. Sterile MQ water was then perfused for 18 h to wash away the detergent and was switched to PBS for 6 more hours.

### Validation of Decellularization

Decellularization was validated macroscopically and microscopically at histological level by collecting whole mount samples from fresh tissue and decellularized scaffolds. Tissues were fixed in 10% formalin/PBS for 20 min and permeabilized with 0.2% Triton X‐100/PBS for 3 h, washed in PBS, stained with 1 µg mL^−1^ DAPI (Thermofisher, USA), 1:500 phalloidin‐Alexa 633 (Thermofisher, USA) in PBS for 3 h in dark, followed by washing in PBS. Single images from the largest optical section (indicated by SHG signal) were acquired on the inverted confocal microscope (Leica SP5‐X, Leica, Germany) with sequential excitation by 405 laser (for DAPI) and white light laser (WLL) (for Alexa‐633) and emission detected by the hybrid detector (HyD S, Leica, Germany). Fluorescence intensity using Fiji software (National Institutes of Health, USA) was quantified per smaller regions of interest (ROIs) of 1024 × 1024 images which included tissue parenchyma. From the raw values was subtracted background fluorescence and values were normalized by the average of intensity per area of control.

### Murine Cell Culturing

4T1‐H2B‐GFP cells were previously generated by stable transfection of 4T1 with a pBOS‐H2BGFP vector (BD Pharmingen, USA)^[^ ^48^ ^]^ and were cultured in Dulbecco's modified Eagle's medium GlutaMAX (DMEM Glutamax; Gibco, USA) with 10% v/v Fetal bovine serum (FBS; Gibco, USA) and 100 U mL^−1^ penicillin‐streptomycin (P‐S; Gibco, USA).

Mouse normal mammary fibroblasts mNF1 were a kind gift from the Erik Sahai laboratory (The Francis Crick Institute, London, UK). They were isolated from normal breast tissue of FVB/n Polyoma Middle T antigen oncogene under the Mouse Mammary Tumour Virus promoter (MMTV‐PyMT) mice and immortalized as described previously.^[^ ^49^ ^]^ Cells were cultured in DMEM high glucose (Gibco, USA) with 10% FBS (Gibco, USA) and 1% v/v Insulin, Transferrin, Selenium Solution (ITS‐G; Gibco, USA), and 100 U mL^−1^ P‐S. KPCmT4 cells were a kind gift from the Tuveson laboratory (Cold Spring Harbor Laboratory, New York, USA). They were isolated from  pancreatic tumors obtained from *Pdx1–Cre* ^+^;*Kras^LSL‐G12D/+^;Trp53^LSL‐R172H/+^* mice of a pure C57BL/6 background as described previously^[^ ^50^ ^]^ and cultured in DMEM Glutamax 10% FBS and 100 U mL^−1^ P‐S.

Primary murine macrophages were generated by flushing the bone marrow from the femur and tibia of C57BL/6 mice followed by red blood cell lysis (BD Biosciences, USA) and density gradient centrifugation on histopaque (1083 g mL^−1^) (Sigma, USA). The middle layer was collected and differentiated into macrophages by incubation for 5 days in DMEM (Gibco, USA) containing 10% FBS (Gibco, USA) and 10 ng mL^−1^ murine macrophage colony‐stimulating factor (M‐CSF; Peprotech, USA). All cells were maintained in a 37 ^*ο*^C humidified atmosphere under 5% CO_2_ and were regularly tested for mycoplasma.

### Human Cell Culturing

T‐cells peripheral blood mononuclear cells (PBMC) from buffy coats were isolated from healthy donors provided by the blood bank at Copenhagen State University Hospital (Rigshospitalet, Denmark) as previously described.^[^ ^51^ ^]^ These experiments were approved by the Ethical Committee of the Hospital Region of the Capital Region. In short, buffy coats were diluted in PBS and PBMC were isolated by density gradient centrifugation on a layer of Lymphoprep (Fresenius Kabi, Denmark) for 30 min at 1200 RPM without breaks. The isolated PBMC were cryopreserved in FBS + 10% dimethyl sulfoxide (DMSO; Sigma Aldrich, USA). Ethical review and approval were not required for the collection of PBMC from human participants, in accordance with Danish legislation and institutional requirements. Written informed consent for participation was not required for this study in accordance with Danish legislation and institutional requirements.

The cells were phenotyped for CD3+ (99%) and cultured in X‐vivo medium (Lonza Biotech, USA) supplemented with 5% human serum (Sigma Aldrich, USA), 100 U mL^−1^ P‐S, and 1000 U mL^−1^ human recombinant interleukin‐2 (Miltenyi Biotech, USA). T‐cells were transduced with a lentivector containing human MAGE‐A3^a3a^ TCR.^[^ ^31^ ^]^ Transduced T‐cells were sorted with melanoma associated antigen‐3 (MAGE‐A3) tetramers (>90%). TCR expression was verified by TCR Vb5.1 monoclonal antibody (>90%). T‐Cells were tested for cytotoxicity against MAGE‐A3 expressing human melanoma cell line FM82 in a 51Cr‐release assay. Human melanoma cells, FM82, were obtained from the European Searchable Tumor Line Database and transduced with a GFP‐Luciferase vector. They were cultured in RPMI 1640 (Gibco, USA) supplemented with 10% FBS (Gibco, USA) and 100 U mL^−1^ PS (Gibco, USA).

Cancer organoids were established from 18G core‐needle biopsies of liver metastasis of colorectal cancer patients, who consented to the organoid protocol, in agreement with the Ethics Committee of the Capital Region of Denmark (number H‐16046103, the Data registry j.nr.: 2012‐58‐004). Excess organoid culture from the cancer organoid protocol was used in these experiments. Biopsies were processed for establishing organoid cultures as previously described.^[^ ^52^ ^]^ Organoids were grown inside Matrigel (R&D Systems, USA) in a basal culture medium [phenol red free DMEM/F12 (Gibco, USA) with P‐S and 10mM HEPES (Sigma, USA), B27 and N2 (Gibco, USA)], supplemented with human Neuregulin‐1 Noggin and R‐spondin (all at 100 ng mL^‐1^; R&D Systems, USA) and maintained in a 37 °C humidified atmosphere with 5% CO_2_.

### Introduction of Cells and Cell Culture in the Bioreactor

Prior to introducing cells, the bioreactor was perfused with cell culture medium for 12 h at a speed of 70 µL min^−1^. The chamber temperature was stabilized by placing over a heating panel at 37 °C (Temperature control unit, Harvard Apparatus, USA). To seed cells the flow was stopped and the four‐way stopcock (BD, USA) on the tubing was directed from the injection point to the bioreactor. A syringe with a cell suspension was introduced into the stopcock entrance. The cells were injected in 1 mL volume at a speed of ≈250 µL min^−1^ (controlled by rotating the syringe plunger), with a maximum of 1 × 10^6^ cells mL^−1^. If more than 1 × 10^6^ cells were injected, successive 1 mL injections were used. Successful injection was confirmed by localizing fluorescent cells inside organ scaffold under a fluorescent microscope. The flow was restarted after injections were finished.

Lung ECM scaffolds were repopulated with 2 × 10^6^ mNF1 via the trachea or 1.5 × 10^6^ 4T1‐H2B‐GFP via the aorta, or a sequential combination of both. Cancer cells were diluted to the final concentration 10^6^ mL^−1^ in serum‐free DMEM Glutamax medium (Gibco, USA). To visualize fibroblasts, cells were pre‐labeled with Vybrant DiD cell labeling solution (Thermofisher, USA) according to the manufacturer's instructions and prepared at 10^6^ mL^−1^ concentration in the full DMEM medium. Cell suspensions were gently mixed with a pipette and loaded into a 1 mL syringe.

For co‐culture bioreactors, mNF1 fibroblasts were cultured in the bioreactor for 1 week, and subsequently 4T1‐H2B‐GFP cells were injected and cultured for 1 week in full cancer cells medium. For single cell line culturing, matching times were chosen—mNF1 for 1 week and 4T1‐H2B‐GFP 1 week.

For experiments modeling immunotherapy in lung ECM scaffolds, first 1 × 10^6^ human FM82‐GFP‐Luc melanoma cells were injected via the trachea and 1 h later 3 × 10^6^ human engineered T‐cells stained with Vybrant DiI cell labeling solution (Thermofisher, USA) were injected via the aorta. Cells were co‐cultured for 24 h under perfusion with T‐cells medium.

For cell culture experiments with KPCmT4 and macrophages in the liver ECM bioreactor, first 1 × 10^7^ KPCmT4 cells were injected and 1 h later 2 × 10^6^ macrophages. Cells were co‐cultured for 24 h under perfusion with the full KPCmT4 medium.

For culturing of patient‐derived cancer organoids, a liver ECM bioreactor was injected with 5 × 10^6^ cells obtained from disaggregated organoids and cultured either in basal medium for 48 h, or in basal medium with growth factors for 1 week (see Results section).

### Monitoring Live‐Cell Imaging

To perform live‐cell imaging, a repopulated ECM scaffold was laid on the optical glass of a bioreactor chamber so that its ventral surface faced the objective. The aspiration tube was rested on the side of the chamber base to create a liquid level of ≈1 mL depth. Culture medium was perfused with a peristaltic pump (Ole Dich, Denmark) or, to minimize drift, by a syringe pump (World Precision Instruments, USA) at 30 µL min^−1^ flow speed.

Bioreactors were monitored with an inverted confocal microscope (Leica SP5‐X, Leica, Germany) with a 2P Ti‐Sapphire laser with tunable wavelength (690–1040 nm) (MaiTai DeepSee laser, Spectra‐Physics, USA). To detect fibrillar collagens by SHG the 2P laser was excited at 870/892 nm and emission at a halfwave detected by a Hybrid detector (HyD2, Leica). Fluorescent signal from GFP+ cells was simultaneously excited by the 2P laser, while the Vybrant DiI (ex: 549) was excited using a WLL (Leica). Sequential images were acquired using a 10× PLAN APO ×0.40 NA dry objective with additional optical zoom. Imaging was done in a single 2D plane (T‐cells with melanoma cells), or in 3D using z‐step of 25 µm (lung bioreactor) (total stack depth = 100–130 µm). A single area of 1024 × 1024 pixels was recorded.

### Live Imaging Analysis and Cell Migration Tracking

Recorded movies were processed with Fiji software (National Institutes of Health, USA). Videos were corrected for drift with Descriptor‐based series registration (2D/3D+t) plugin (by Stephan Preibisch). ROIs were analyzed with Trackmate Fiji plugin.^[^ ^53^ ^]^ Track displacement and maximum speed were quantified using the plugin.

### Immunofluorescence Staining of Organoids

Isolation from Matrigel and staining of the organoids was performed as previously described.^[^ ^54^ ^]^ Briefly, organoids were washed with PBS before incubation in cell recovery solution (Corning, 354253) for 1 h on a horizontal shaker at +4 °C. Then the suspension from wells was pelleted (70 g, 4 min, +4 °C) and resuspended in 4% paraformaldehyde (PFA; Thermofisher, USA. Fixation lasted for 45 min on wet ice (carefully resuspended in between) and was stopped by addition of 0.1% v/v Tween‐20 (Sigma, USA) in PBS for 10 min. After washes organoids were stained with either pancytokeratin (rb, ab9377, abcam)—1:200 or vimentin (rb, ab92547, abcam)—1:200 primary antibodies, followed by secondary antibody (donkey anti‐rabbit 488 (Thermofisher, USA)—1:500), DAPI (1 µg mL^−1^), and phalloidin 633 (1:500). Organoids were embedded for imaging as previously described.^[^ ^54^ ^]^

### Immunofluorescence Staining of Bioreactors

For imaging fixed bioreactor samples, repopulated scaffolds were taken out of the bioreactor box, rinsed in PBS to remove medium, and immersed in 10% formalin/PBS for 20 min, followed by 3 washes in PBS. Cut samples were stained as previously described.^[^ ^47^ ^]^ The samples were incubated in 0.2% TritonX‐100/PBS for 30 min if permeabilization was required. Otherwise, samples were next blocked overnight in a freshly made 3% w/v bovine serum albumin (BSA), 6% v/v donkey serum (Jackson ImmunoResearch)/PBS. After washing once with PBS the samples were incubated in primary antibody solution with 3% v/v donkey serum/PBS. 20–24 h later, the tissues were washed (5 times 1 h) with 0.05% v/v Tween‐20/PBS solution, followed by incubation in a secondary antibody solution with 3% w/v donkey serum overnight in the dark. All steps were performed at room temperature (RT). Finally, tissues were washed (3 times 1 h) with 0.05% v/v Tween‐20/PBS solution and for storage were placed in 1% v/v P‐S and 0.3 µm sodium azide (Sigma, USA)/PBS solution at +4 °C shortly until imaging.

Staining of the bioreactor with KPCmT4 and macrophages was done as following: bioreactor was fixed with 4% PFA/PBS for 30 min at RT. Washed with PBS, permeabilized 0.5% TritonX‐100/PBS for 1 h at RT, and washed in PBS (3 times 5 min). Blocked with 3% BSA/PBS for 1 h at RT, washed with PBS. Primary antibody added in 1% BSA/PBS, incubated at +4 °C overnight, and washed with PBS. Secondary antibody with DAPI and phalloidin was added in 1% BSA/PBS and incubated at RT for 2 h in the dark, washed with PBS, and stored in PBS at +4 °C shortly until imaging.

Primary antibodies used: collagen IV (gt, AB769, Millipore)—1:100; ki‐67 (rb, D3B5, 12202S, Cell Signaling Technology)—1:300; F4/80 (rt, #14‐4801‐82, clone: BM8, eBiosciences)—1:100.

Secondary antibodies used: donkey anti‐goat Alexa‐594 1:1000 and donkey anti‐goat Alexa‐555 1:1000; donkey anti‐rabbit 488—1:1000; goat anti‐rat Alexa 488—1:500 (Thermofisher, USA). Some samples were additionally stained with DAPI to visualize nuclei —1 µg mL^−1^ and/or phalloidin Alexa‐633 to visualize F‐actin—1:500 for 30 min at RT.

### Imaging of Fixed Samples

For imaging, bioreactor samples were placed on a glass‐bottom dish (MatTek, 35‐mm) immersed in a drop of a storage solution. Images for evaluation of the ECM remodeling were acquired on the inverted confocal microscope (Leica SP5‐X, Leica, Germany). 2P laser was used for simultaneous SHG and GFP excitation, while WLL was used to excite Alexa 555, Alexa594, phalloidin‐Alexa633 or Vybrant DiD (ex: 644 nm), using sequential excitation. Objective HCX PL APO lambda blue 20×, 0.70 NA IMM UV was used. Several stacks per sample were acquired with 1024 × 1024 pixels resolution, at 100–200 Hz, z‐step of 7 µm.

Images from the liver bioreactors repopulated with KPCmT4, macrophages and from the organoids, bioreactors with growth factor reduced organoid cells were acquired on an inverted confocal microscope (Leica SP8, Leica, Germany) using WLL with a HC PL APO CS2 20×/0.75 IMM water objective.

### Lung ECM Bioreactor Morphological Evaluation

To evaluate complex structural changes in the lung ECM bioreactors, surgical pathologists performed qualitative histopathological assessment on blinded imaging sets, consisting of maximum intensity projected stacks of images. Normal, “healthy” tissue ECM structure was defined by images from cell‐free bioreactors perfused with culture medium. Abnormal, “diseased” ECM structure was different from normal, healthy ECM and had changes in collagen IV compatible with those produced by metastatic, invasive lung tumors.

### Lung Collagen IV Remodeling Quantification

For quantitative analysis of ECM remodeling were used maximum intensity projection images of lung parenchyma immunostained for collagen IV taken from several ROIs (354 × 322 pixels). Smaller images allowed to focus on alveolar parenchyma without large vessels, pleura, large airways. Images were analyzed in TWOMBLI plugin (version December 2020)^[^ ^55^ ^]^ in Fiji software after an optimal mask (binary image from col IV) and other parameters were established: contrast saturation (0.35), line width (17), and minimum branch length (10). Endpoints number was normalized by total length of branches per ROI.

### Protein Extraction from Plastic, Tissue, and Bioreactor Scaffolds

For kinase profiling, cell pellets from 4T1‐H2B‐GFP cells cultured on plastic for 1 week and aorta‐injected 4T1 lung bioreactors cultured for 1 week in the full medium were collected in addition to the in vivo samples. Samples were stored at −80 ° C until all were collected and then defrosted and minced using scalpels, after which Mammalian Protein Extraction Reagent (M‐PER; Thermofisher) containing EDTA‐free protease inhibitors (1:100) and phosphatase (1:100) inhibitor cocktails (Halt; Thermofisher) were added to lyse cells (60 min, 4 °C, 500 rpm [Biometra TSC Thermoshaker]). Lysed samples were centrifuged (10 min, 4 °C, 16 000 g), the supernatant was collected, and protein concentration was determined by Bradford assay.

### Kinase Profiling Sample Preparation

Kinase profiling was performed using tyrosine kinase PamChips processed on the Pamstation12 instrument (Pamgene, Netherlands), as previously described.^[^ ^56^ ^]^ Protein samples (7 µg) prepared with a FITC‐conjugated anti‐phosphotyrosine antibody (PY20) were dispensed onto a porous membrane on the PamChip, which contained peptides with known phosphorylation sites. The Pamstation12 pumps each sample through the membrane, allowing kinases in the sample to actively phosphorylate substrates on the PamChip, which was detected with a built in CCD camera.

### Kinase Profiling Analysis

Raw images were analyzed using BioNavigator software (Pamgene, Netherlands), and relative peptide signal intensities between samples were quantified using a *t‐*test (Table S1, Supporting Information). In addition, prediction of kinases responsible for altered phosphorylation between conditions was analyzed using the upstream kinase analysis app (2018 version; PamGene) and was based on kinase‐substrate relationships reported in multiple databases. Summary of the output values—the mean kinase statistic (a proportional measure of activity for a kinase when evaluating a set of peptides that were linked to that kinase across replicates) and the combined score (significance across replicates) are listed in the Table S2, Supporting Information.

### Statistical Analysis

Graphs were plotted and statistical analysis was performed in GraphPad Prism software (version 7.4). Data were normalized when mentioned in the text or figure legends. Particular sample size, datapoints format, and statistical tests are mentioned in the matching figure legends.

## Conflict of Interest

The authors declare no conflict of interest.

## Author Contributions

M.R. and A.E.M.‐G. contributed equally to this work. A.E.M.‐G. conceived the project and developed it together with J.T.E., M.R., and C.D.M. A.E.M.‐G., M.R., and J.T.E. designed all experiments. A.E.M.‐G. designed the bioreactor and surgical decellularization procedure. M.R., A.R.D.J., and S.R.N., assisted by A.E.M.‐G., prepared and introduced cells into bioreactors. A.E.M.‐G., M.R., and A.R.D.J. maintained bioreactors. A.E.M.‐G. and M.R. did animal experiments. A.E.M.‐G., M.R. with C.D.M. assistance, performed live‐cell imaging. M.R. performed cell migration tracking analysis. E.R.H. and A.R.D.J. performed kinase profiling and analysis. M.R., R.R., O.W., and S.R.N. performed staining of bioreactors. M.R., C.D.M., and A.E.M.‐G. imaged and analyzed stained bioreactors. C.B.B. and T.H.J. (certified pathologists) provided expertise on lung histology and supported ECM analysis. S.R.N. provided macrophages and KPC cells. M.I. and P.T.S. provided human melanoma cells and engineered T‐cells. K.W.Z., D.S.G., and M.C. established patient‐derived colorectal organoids and prepared cells for bioreactor seeding. K.R., I.S., M.H., and U.L. conducted preclinical program for cancer precision medicine and provided access to patient biopsies for establishing organoid cultures. M.R., A.E.M.‐G., J.T.E., R.R., and E.R.H. wrote the manuscript. J.T.E. and A.E.M.‐G. supervised the project.

## Supporting information

Supporting Information

Supplemental Movie 1

Supplemental Movie 2

Supplementary Table 1

Supplementary Table 2

## Data Availability

The data that supports the findings of this study are available in the supplementary material of this article. The other data that support the findings of this study are available from the corresponding author upon reasonable request.
