# Supplementary material for: Modeling Metastatic Colonization in a Decellularized Organ Scaffold‐Based Perfusion Bioreactor
Source: Adv Healthc Mater. 2021 Nov 17;11(1):2100684. doi: 10.1002/adhm.202100684 (PMC11469127; doi:10.1002/adhm.202100684)
Supplement: Supplementary file 1 — Supporting Information [file ADHM-11-2100684-s005.pdf]

ADVANCED HEALTHCARE MATERIALS

Supporting Information

for *Adv. Healthcare Mater.*, DOI: 10.1002/adhm202100684

Modeling metastatic colonization in a decellularized organ scaffold-based perfusion bioreactor

Maria Rafaeva[#], Edward R. Horton, Adina R.D. Jensen, Chris D. Madsen, Raphael Reuten, Oliver Willacy, Christian B. Brøchner, Thomas H. Jensen, Kamilla Westarp Zornhagen, Marina Crespo, Dina S. Grønseth, Sebastian R. Nielsen, Manja Idorn, Per Thor Straten, Kristoffer Rohrberg, Iben Spanggaard, Martin Højgaard, Ulrik Lassen, Janine T. Erler, Alejandro E. Mayorca-Guiliani[#]**

Title

Modeling metastatic colonization in a decellularized organ scaffold-based perfusion bioreactor

Maria Rafaeva[#], Edward R. Horton, Adina R.D. Jensen, Chris D. Madsen, Raphael Reuten, Oliver Willacy, Christian B. Brøchner, Thomas H. Jensen, Kamilla Westarp Zornhagen, Marina Crespo, Dina S. Grønseth, Sebastian R. Nielsen, Manja Idorn, Per Thor Straten, Kristoffer Rohrberg, Iben Spanggaard, Martin Højgaard, Ulrik Lassen, Janine T. Erler, Alejandro E. Mayorca-Guiliani[#]**

# These authors contributed equally

** Corresponding authors*

((Please insert your Supporting Information text/figures here. Please note: Supporting Display items, should be referred to as Figure S1, Equation S2, etc., in the main text…)

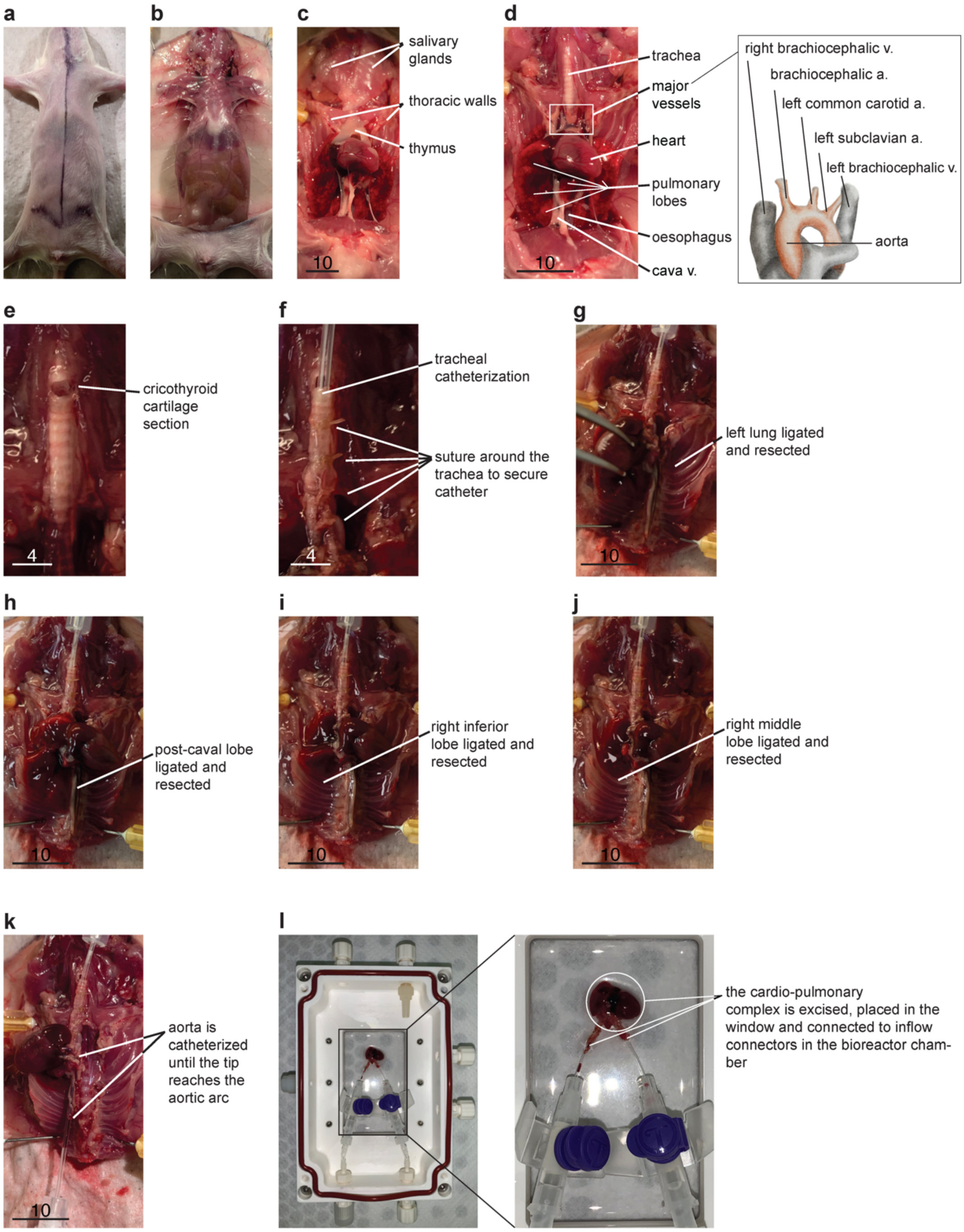

Supplementary figure 1. Step-by-step surgical instructions for the lung bioreactor. a-k, Detailed surgical steps to isolate the superior pulmonary lobe. **l,** Placement of the excised lung lobe with the heart in the bioreactor chamber. All scale bars in millimeters.

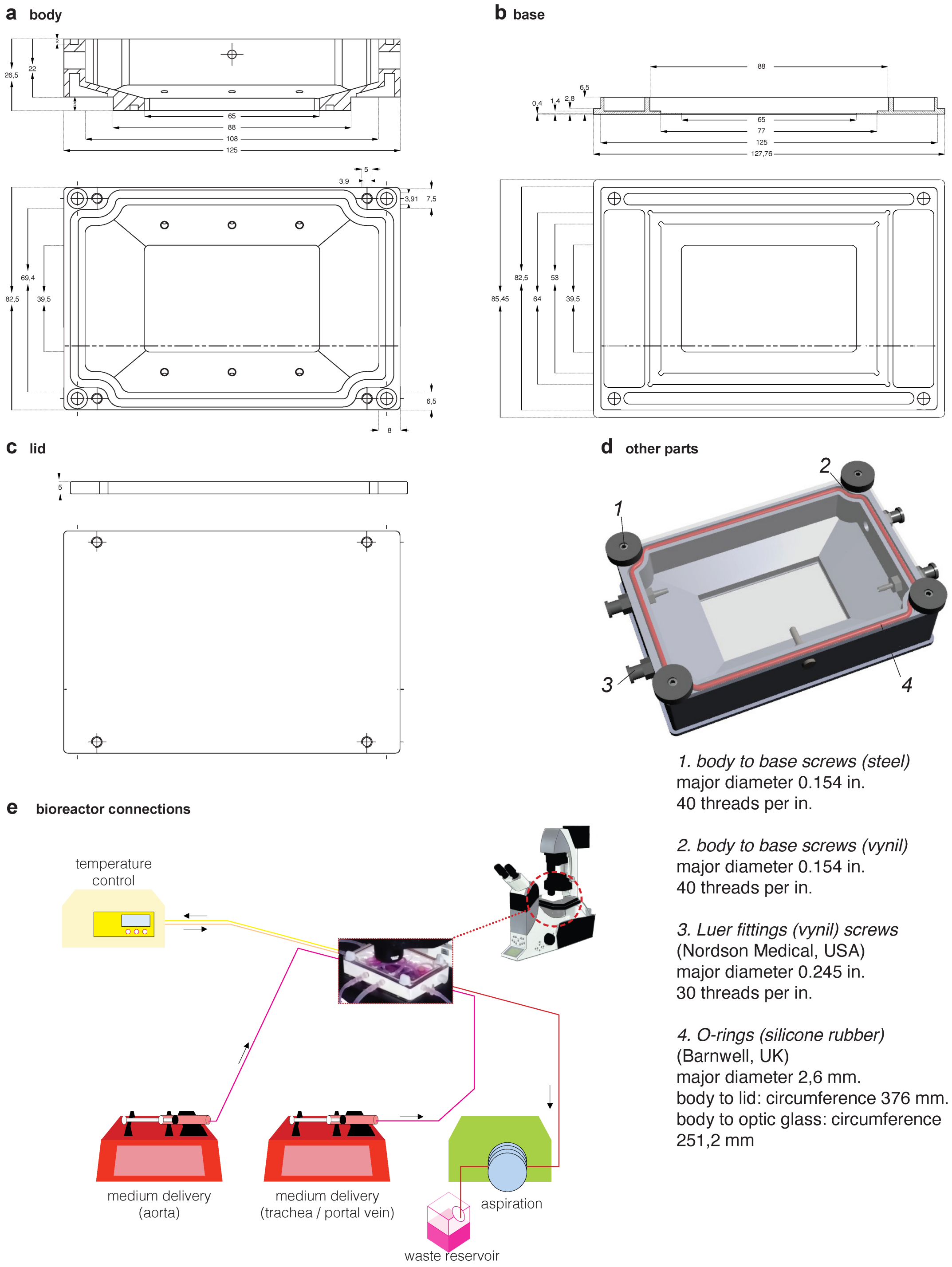

Supplementary figure 2. Design of the bioreactor chamber and its perfusion set-up for live cell imaging. Detailed schematic of the **a,** chamber body. **b,** chamber base. **c,** lid. **d,** 3D rendering of the chamber. **e**, Schematic of the bioreactor chamber perfusion during live-cell imaging at the microscope.

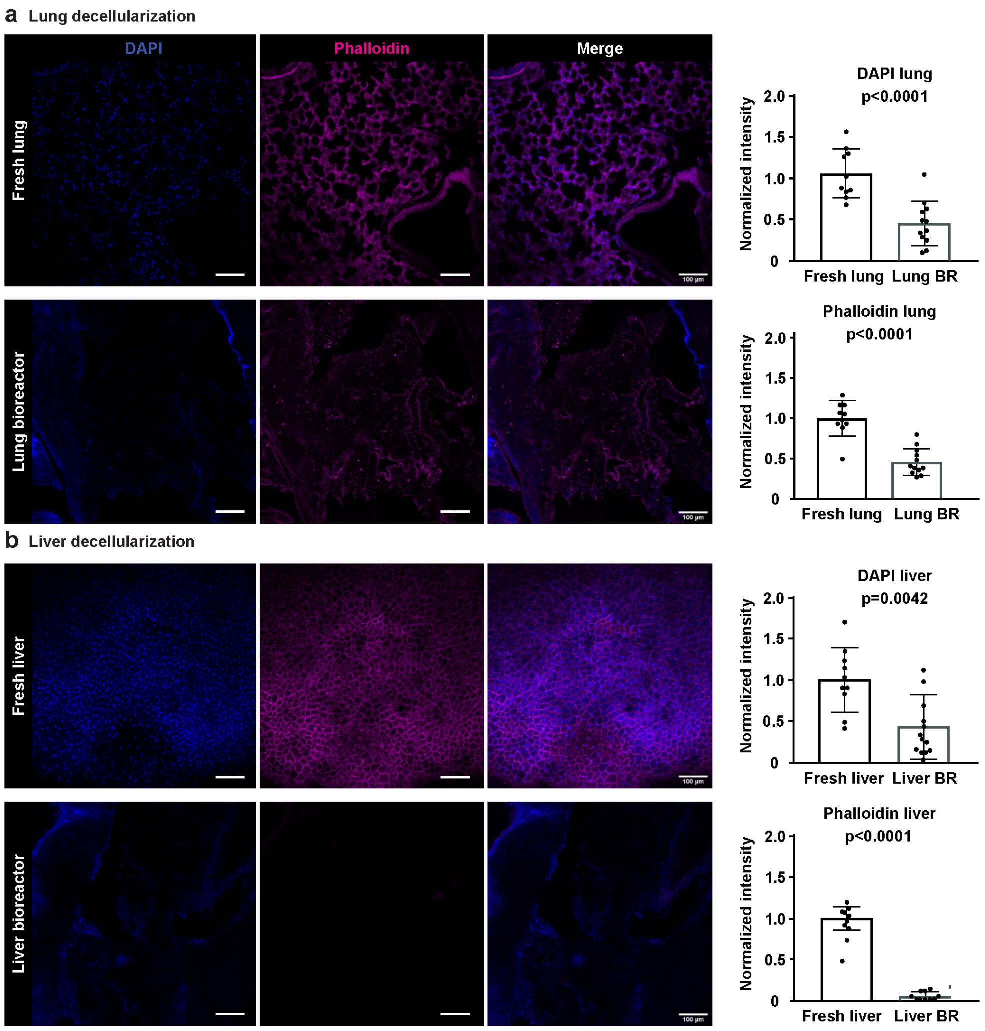

Supplementary figure 3. Validation of decellularization at histological level. a. Representative images and quantification of whole-mount samples from fixed fresh lung and lung bioreactor scaffold stained with DAPI (for DNA) and phalloidin (for β-actin). **b.** Representative images and quantification of whole-mount samples from fixed fresh liver and liver bioreactor scaffold stained with DAPI (for DNA) and phalloidin (for F-actin). Unpaired t-test. n=3 samples per condition. All scale bars in microns.

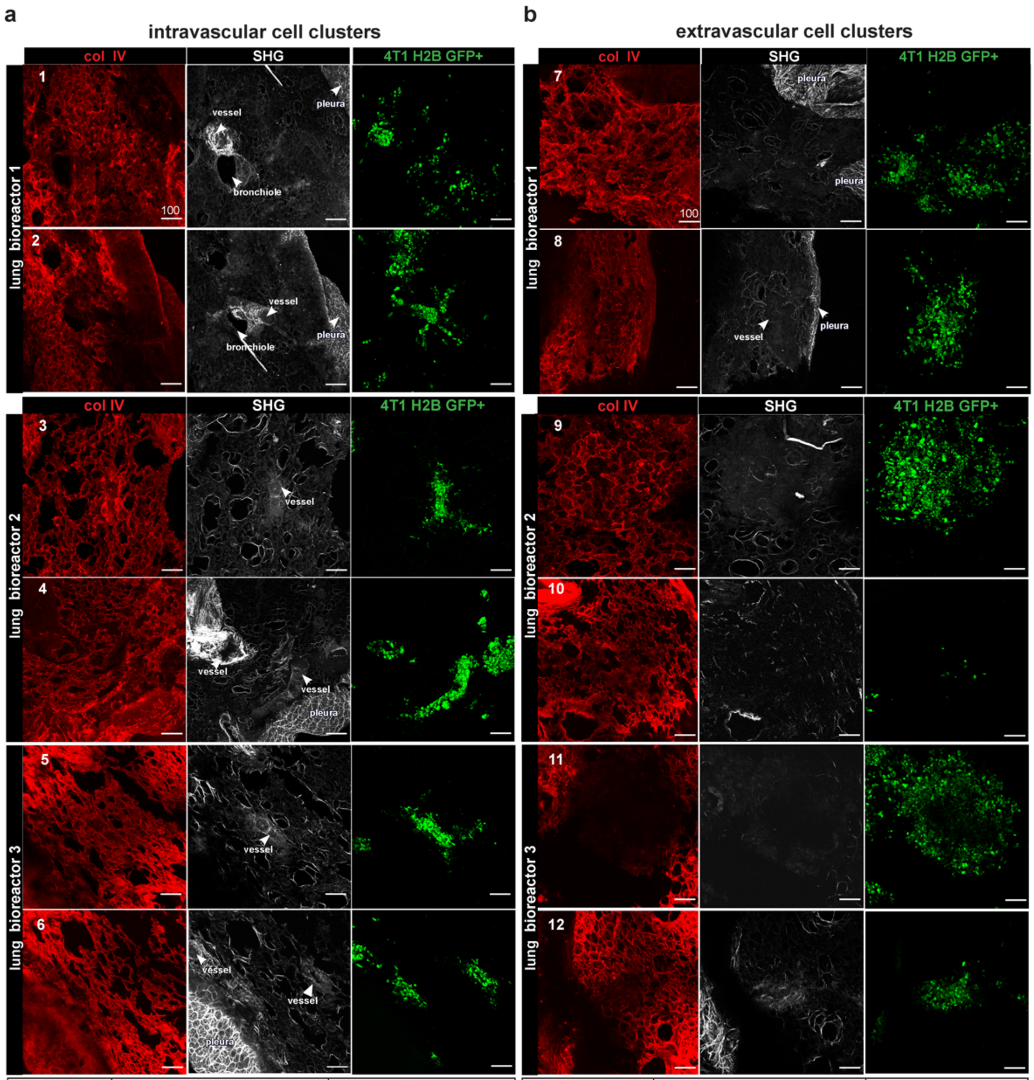

image	col IV	SHG
1	abnormal. disturbed col IV structure in cell bearing areas	abnormal. signal increase in cell bearing areas
2	abnormal. remodeled col IV structure in cell-bearing areas	abnormal. signal increase in cell bearing areas
3	abnormal.disturbed col IV structure in cell bearing areas	abnormal. increased signal in cell-bearing areas
4	normal	normal
5	normal	normal, but signal increase in vessel lumen
6	normal	normal

image	col IV	SHG
7	abnormal. septal destruction in cell-bearing areas	normal
8	abnormal. remodeled col IV structure	abnormal. weak signal in vessel wall
9	abnormal. local loss of col IV structure	abnormal. increased signal in cell-bearing areas
10	normal but slightly disturbed col IV areas	normal
11	abnormal. erased col IV structure	abnormal. no normal architecture present.
12	abnormal. weak signal in cell-bearing areas	abnormal. no normal architecture present

Supplementary figure 4. Morphological evaluation of ECM remodeling in lung bioreactors with cancer cells. 4T1-H2B-GFP cells cultured in lung bioreactors for 1 week. ECM is visualized by col IV antibody staining and by SHG imaging of fibrillar collagens. n=3 bioreactors. **a**. Intravascular cell cluster areas. **b**. Extravascular cell clusters area. **c.**

Summary table with the statement from pathologists about col IV and fibrillar collagen (SHG) morphology. All scale bars in microns.

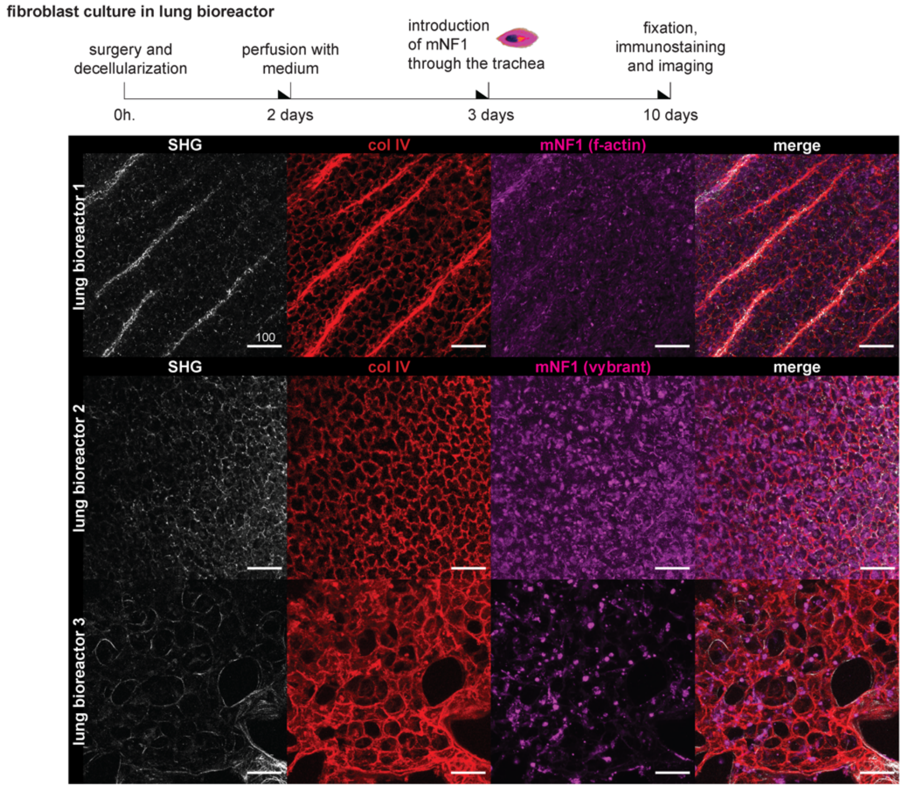

image	col IV	SHG
bioreactor 1	normal	normal
bioreactor 2	normal	normal
bioreactor 3	normal	normal

Supplementary figure 5. Morphological evaluation of ECM remodeling in lung bioreactors with normal fibroblasts. Lung ECM scaffold structure and mNF1 localization after introduction via trachea and culturing for 1 week. n=3 bioreactors. ECM is visualized by col IV antibody staining and by SHG imaging of fibrillar collagens. mNF1 are stained by phalloidin (F-actin) or a cell dye (Vybrant). Summary table shows the statement from pathologists about col IV and fibrillary collagen (SHG) morphology. All scale bars in microns.

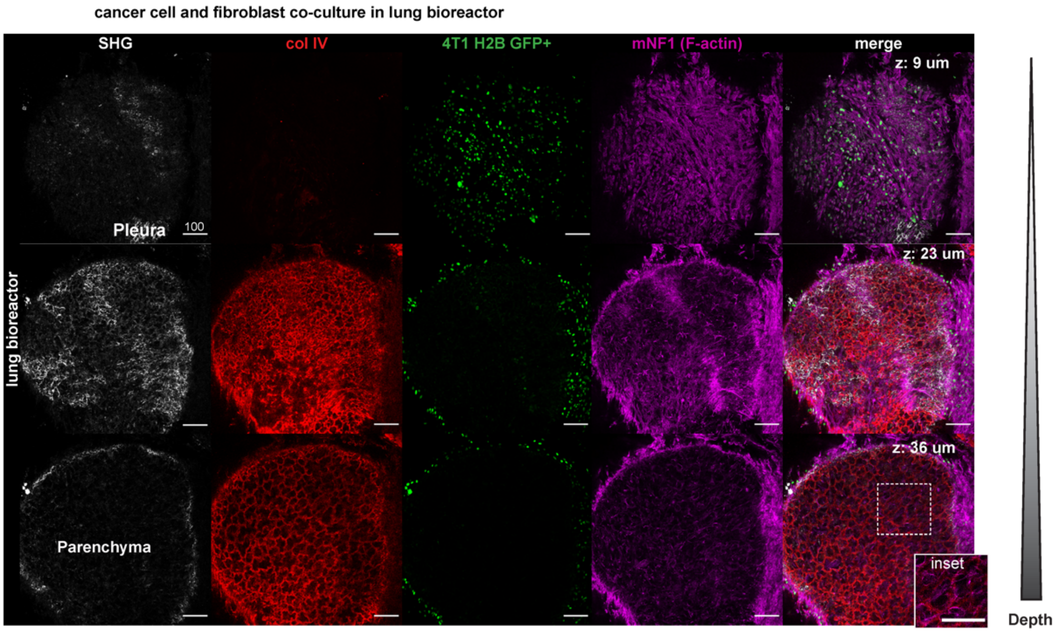

Supplementary figure 6. Co-culture of normal fibroblasts with cancer cells in lung bioreactor. a. Deconstruction of a z-stack showing spatial distribution of cells. z-stack depth is noted in the images. Inset shows enlarged area with fibroblasts spreading along alveolar septa. Cancer cells are discriminated by H2B-GFP nuclei, both cell types are stained with phalloidin. All scale bars in microns.

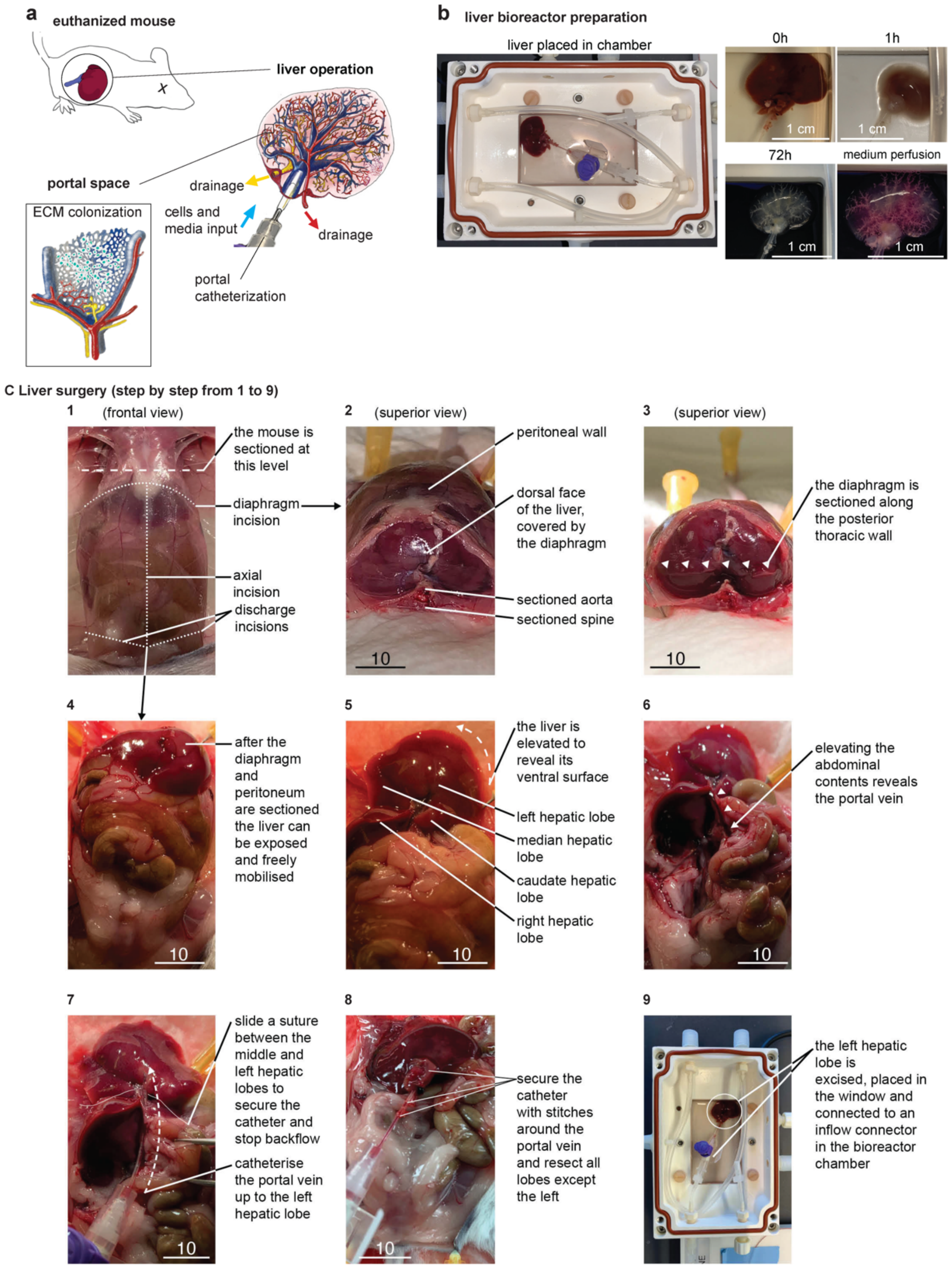

Supplementary figure 7. Step-by-step surgical instructions for a liver bioreactor scaffold. **a,** liver bioreactor schematic. **b,** liver decellularization timeline **c, Liver surgery. 1-8,** Detailed surgical steps to isolate the left hepatic lobe. **9,** Placement of the lobe in the bioreactor chamber. All scale bars in millimeters except when indicated.

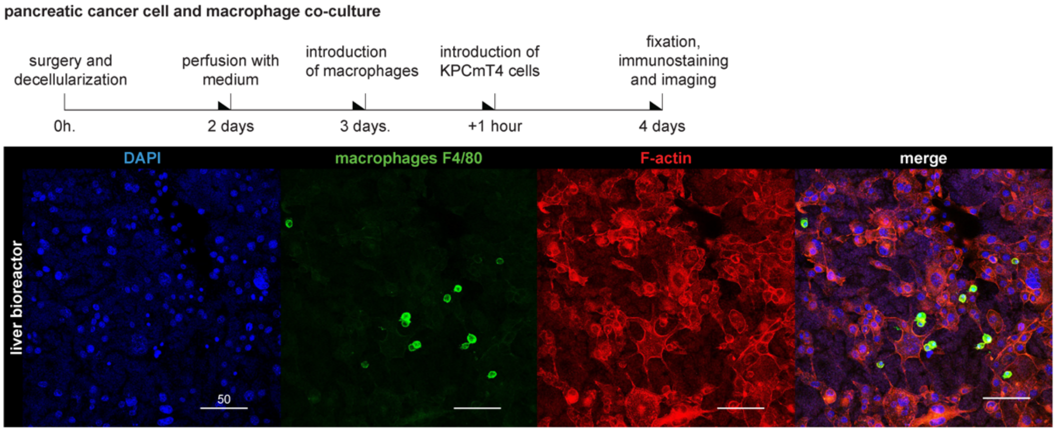

Supplementary figure 8. Co-culture of pancreatic cancer cells with macrophages in the liver bioreactor. Schematic of preparation and representative image of liver bioreactor populated with primary mouse macrophages and KPCmT4 pancreatic cancer cells. Macrophages are visualized by staining against F4/80 cell surface marker. Both cell types are stained by DAPI and phalloidin. All scale bars in microns.

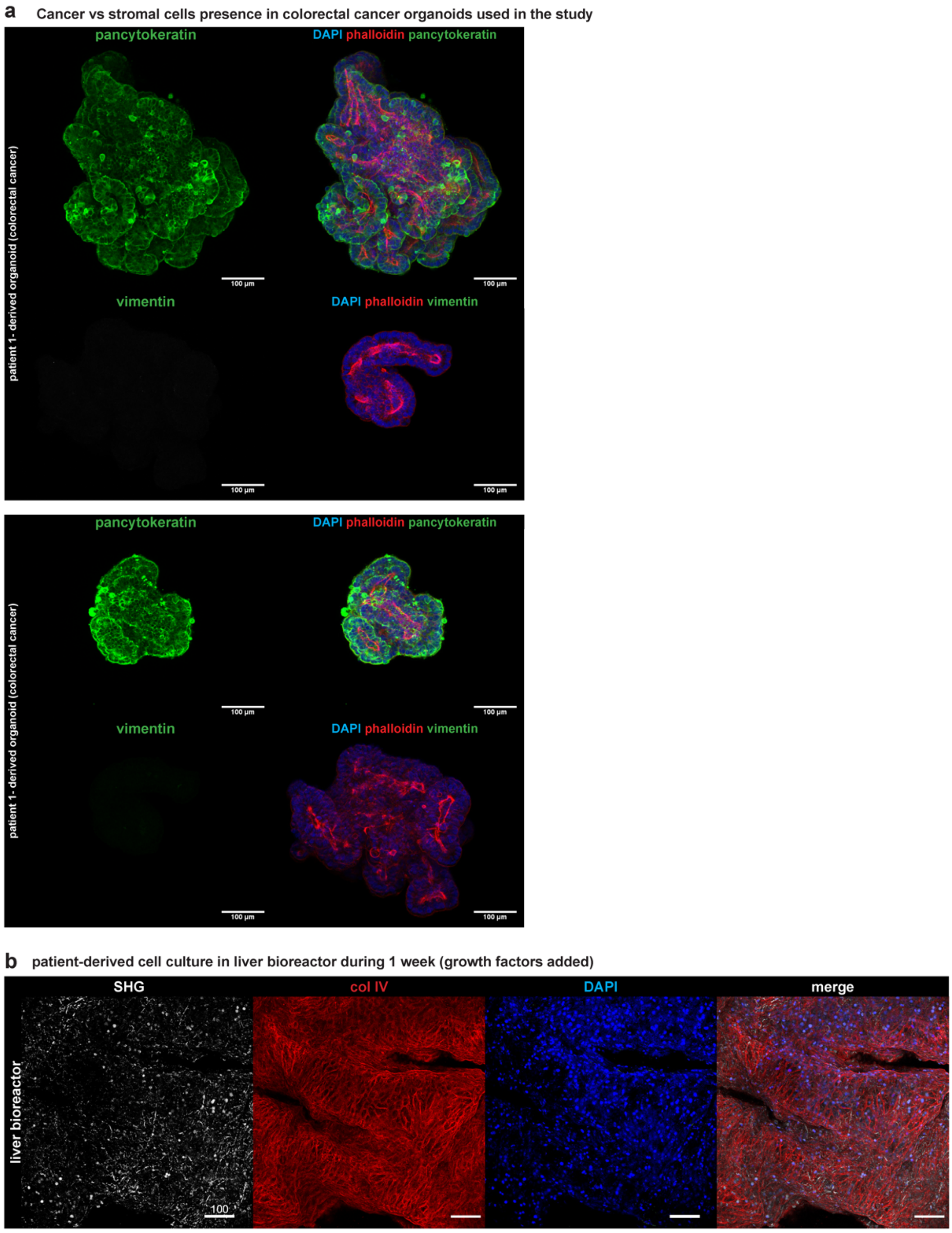

Supplementary figure 9. Patient-derived organoids cells populating the liver bioreactor. Organoid cells suspension was injected through the portal vein and cells were cultured in the presence of growth factors for a week. DAPI shows cell nuclei. All scale bars in microns.

Other types of Supporting Information, e.g., multimedia files, raw data, code, etc., should be provided separately, with large files provided in a zip file. If you experience difficulty uploading large files, please contact the editorial office for assistance.
